# Insight into Current Practices of Community Pharmacists in Topical Corticosteroid Prescribing and Counseling: Cross-Sectional Survey Study from Saudi Arabia

**DOI:** 10.3390/healthcare12141425

**Published:** 2024-07-17

**Authors:** Sawsan M. Kurdi, Ahmad Alamer, Arjwan Alqarni, Sara AlQahtani, Shahad AlKahlah, Fawaz M. Alotaibi, Ibrahim M. Asiri, Haytham A. Wali

**Affiliations:** 1 Department of Pharmacy Practice, College of Clinical Pharmacy, Imam Abdulrahman Bin Faisal University, Dammam 34221, Saudi Arabia; 2190003018@iau.edu.sa (A.A.); 2190002926@iau.edu.sa (S.A.); 2210040082@iau.edu.sa (S.A.); fmalotaibi@iau.edu.sa (F.M.A.); imasiri@iau.edu.sa (I.M.A.); 2 Department of Clinical Pharmacy, Prince Sattam Bin Abdulaziz University, Alkharj 16273, Saudi Arabia; aa.alamer@psau.edu.sa; 3 Pharmacy Practice Department, College of Clinical Pharmacy, King Faisal University, Al-Ahsa 31982, Saudi Arabia; hwali@kfu.edu.sa

**Keywords:** topical corticosteroids, counseling, dispensing, community pharmacists

## Abstract

Background: Topical corticosteroids are commonly used to treat several skin conditions, most notably atopic dermatitis. Many studies have found that patients lack knowledge about the safety, potency, and appropriate use of topical corticosteroids. This can be due to ineffective education by pharmacists and other healthcare providers. This study aims to evaluate the appropriateness of dispensing and counseling practices of community pharmacists towards topical corticosteroids in Saudi Arabia. Methods: A cross-sectional survey study was conducted in Saudi Arabia among 418 community pharmacists from different regions of Saudi Arabia. Data were collected using a validated questionnaire that covered community pharmacists’ sociodemographic information, their perceptions of patient knowledge about topical corticosteroid use, and their dispensing and their counseling practices, in addition to their perceived barriers to counseling. Results: The majority of the participating community pharmacists were Saudi (57.4%), female (66.7%), holding a bachelor’s degree (63.4%), and full-time workers (91.1%). Most of the time, community pharmacists counseled patients on the frequency of application per day and the duration of treatment (75.8% and 74.8%, respectively). The median counseling practice score was 17, with an IQR of 14–21. The main barrier to counseling was lack of time (33.7%). Only 15% of community pharmacists accurately identified all scenarios that necessitate medical referrals. Dry skin, itchiness, and irritation were the most common side effects reported by community pharmacists for patients to complain about (69.4%). Most pharmacists agreed that misuse is the most likely cause of topical corticosteroid adverse drug events (53.7%), followed by medication overuse, such as patient self-treatment (48%). Conclusion: Community pharmacists counseling practices to their patients about the use of topical corticosteroids require improvement. Continuing education and hands-on training are needed for community pharmacists regarding counseling about topical corticosteroids use.

## 1. Introduction

Topical corticosteroids (TC) are considered one of the most commonly used in practice by dermatologists to treat several medical conditions, like atopic dermatitis, psoriasis, and many other skin diseases [1]. Thus, TC is considered safe and effective if properly used in consultation with a physician or pharmacist [2,3]. Moreover, TC has anti-inflammatory, antiproliferative, vasoconstrictive, and immunosuppressive characteristics and has various potencies, each of which varies in the degree of vasoconstriction and harm [4,5]. However, the inappropriate use of TC may expose patients to preventable side effects. When topical corticosteroids are used properly, side effects are rare [6]. However, prolonged treatment may cause skin atrophy, hypertrichosis, acneiform lesions, purpura, and telangiectasia [2]. In addition, patients treated with TC are most concerned with skin thinning (atrophy) and systemic absorption [7,8,9,10]. The fear of side effects has led to corticosteroid phobia, which, despite all attempts to prove otherwise, is a key concern for most patients applying corticosteroids topically [10]. Several studies have shown that TC phobia is an outcome of a mix of fear, beliefs, and behavioral aspects, which are the primary factors contributing to TC non-adherence [9,10,11].

Topical corticosteroid phobia is also prevalent and noticeable among pharmacists and other healthcare professionals who warn patients against using topical corticosteroids regularly and emphasize their negative side effects [8,9,10,11,12,13]. Such negative behavior is concerning, given that neither the patient nor the prescriber is certain of the appropriate amount of topical medication to be applied [14]. Although the Saudi Food and Drug Authority [15] regulated TC and mandated its dispensing by an official prescription, a study conducted by Yasmeen et al. claimed that TC can be easily purchased without a prescription in community pharmacies in the Kingdom of Saudi Arabia [16]. Therefore, to properly administer topical corticosteroids, patients rely heavily on the advice provided by their doctors and product specifications. Although warning labels are recommended and appropriate for presenting major side effects for patients, such a practice will not take into account the fact that some patients will take it negatively, impacting TC use. This complicates matters, as these instructions are subjective and unclear, suggesting potential risks if not adhered to [11,17].

Several studies have reported that a high percentage of patients experience poor knowledge of TC safety, potency, and appropriate use. This could be due to insufficient education provided by pharmacists and other healthcare providers [18,19]. Smith et al. found, in a sample of Australian dermatologists, that pharmacists were the most typical source of misinformation leading to steroid phobia [11]. According to some studies, topical corticosteroid phobia may negatively impact patients and parents of children with atopic dermatitis and their adherence to therapy by up to 80.7% [10,20,21].

Another study discovered that a significant number of patients received topical corticosteroid prescriptions or counseling from pharmacists who contradicted dermatologists’ instructions, highlighting a gap in interprofessional collaboration within the practice [22]. This discrepancy in topical corticosteroid instructions has been significantly associated with topical steroid phobia, patient confusion, and delayed treatment initiation [22]. As a result, interprofessional collaboration and consistent information provided by both dermatologists and pharmacists are required for optimal patient care [22,23]. This requires healthcare providers to be knowledgeable about TC and its use. While there is a limited understanding of the correlation between the knowledge and counseling attitudes of community pharmacists, the quality of counseling and the knowledge possessed by pharmacists are essential for the effective and safe application of topical corticosteroids in patients [21,24].

Community pharmacists, also known as retail pharmacists in some countries, play an important role in primary healthcare. Community pharmacists, by education, have the capability to offer various services, including authorizing medication prescriptions, dispensing medications, assessing medication regimens, providing patient counseling to achieve therapeutic outcomes, overseeing therapeutic outcomes, and identifying and resolving concerns related to therapy [25,26]. The most important roles of community pharmacists include reinforcing messages and ensuring that the patient recognizes and understands how to use topical corticosteroids. These roles arise because they have the best opportunity to provide instructions to patients [27,28], as well as to prevent topical corticosteroid abuse by avoiding inappropriate refills [29]. Moreover, a previous study examined community pharmacists’ knowledge regarding topical corticosteroids and found gaps in their knowledge [21], proving that pharmacists are a source of misinformation. Another study carried out in Malaysia revealed that, while community pharmacists possess good knowledge, there is room for improvement in the counseling services they provide [24].

To the best of our knowledge, no study has investigated the counseling and dispensing practices of community pharmacists in Saudi Arabia towards topical corticosteroids. Therefore, this study aimed to assess the appropriateness of counseling and dispensing practices of community pharmacists towards topical corticosteroids in Saudi Arabia.

## 2. Methodology

### 2.1. Study Setting, Design, and Participants

This cross-sectional survey was conducted in community pharmacies in Saudi Arabia. Only licensed pharmacists and technicians working in a community pharmacy setting were eligible to participate in the survey. The study was conducted between January and June 2023, using social media and email and by visiting pharmacies. The study was approved by the Institutional Review Board (IRB) committee at Imam bin Abdulrahman University (IAU) (IRB# 2022-05-430). Participants provided informed consent prior to completing the survey and were able to retract it at any time. All study data were managed under strict confidentiality with no identifiable information.

### 2.2. Validated Questionnaire

The original survey was developed with face and content validity validated by a group of researchers from Korea who employed the knowledge–attitude–practice (KAP) model to construct their instrument [24]. The KAP is a quantitative method that uncovers potential misconceptions acting as obstacles to behavioral change and reveals a disparity between knowledge and actual practice. The survey was subsequently adapted for our study’s objectives, drug names, and professional practice in Saudi Arabia and shared with five community pharmacists possessing relevant experience in the field to gather their feedback.

Based on their feedback, the survey was modified to guarantee that it accurately captured pertinent information and was easily comprehensible to the study population. The finalized survey was subsequently distributed to the study population, ensuring that it accurately mirrored the subject matter and met the needs of the research project.

### 2.3. Sample Size Calculations

Using the online Raosoft^®^ sample size calculator (Raosoft Inc., Seattle, WA, USA), aiming for a 50% response in the surveyed items with a confidence interval of 95% and 5% allowable error, the calculated minimum sample size of 303 precipitants was sufficient for this study.

### 2.4. Statistical Analysis

The data were summarized using descriptive statistics using the Jamovi project (2023) version 2.3. Depending on the variable type, medians with interquartile ranges were used for continuous variables, and counts with percentages were used for categorical variables. Since previous publications have already demonstrated the usefulness of the questionnaire in identifying knowledge and behavior [24], our focus was on describing the current practices in Saudi Arabia. The counseling practice score was formulated from the frequencies of counseling practices of topical steroids. A participant’s score could range from 11 to 33, depending on their counseling practice. They received one point if they did not explain this domain of counseling all the time, 2 points if they explained it half of the time, and 3 points if they explained it all the time. The distribution of the score was illustrated via visualization and descriptive statistics (median and interquartile range). The score was treated as an ordinal variable. Then, the exploratory predictive analysis fitted an ordinal regression to explain the association between a formulated counseling score and baseline characteristics. The odds ratio was reported, along with their 95% confidence intervals (CI), for interpretability. Odds of >1 indicated a higher counseling score associated with the baseline characteristic. Odds < 1 indicated a lower counseling score associated with the baseline characteristic. The significance level was set at alpha < 0.05.

## 3. Results

Of the 418 respondents who completed the survey, 279 (66.7%) were female, and 139 (33.3%) were male. The median age of the participants was 29 years (interquartile range (IQR): 26–34), with a median of 3 years of experience as a community pharmacist. The participants were from different regions of Saudi Arabia, with the majority being from the western province (29.9%), followed by 25.8% from the eastern province. Most participants worked in chain pharmacies (84.4%), were full-time employees (91.1%), and held bachelor’s degrees (63.4%) (Table 1).

### 3.1. Dispensing Practices of Topical Corticosteroids

Table 2 provides insight into the frequency of dispensed topical steroids based on the type, with the highest frequency dispensed as steroid-only agents (42.3%), followed by steroid–antibiotics or steroid–antifungal combination agents (33.7%). Pharmacists reported selling a median of 30 topical corticosteroid prescriptions monthly, with 25 non-prescription topical corticosteroids.

### 3.2. Counseling Practices on the Use of Topical Corticosteroids and Its Barriers

**Figure 1:**
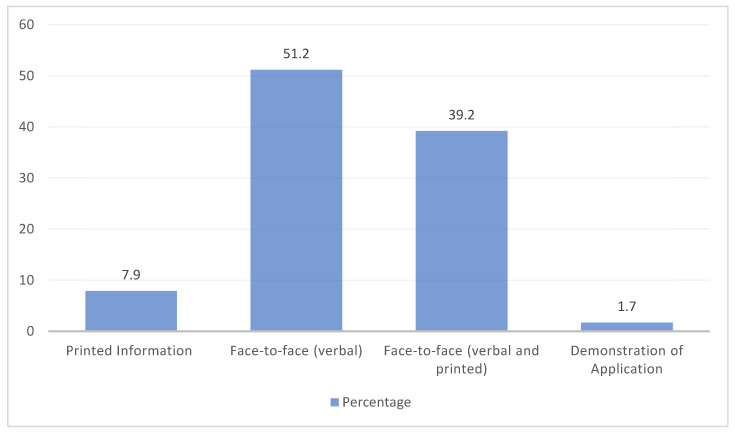
Frequency of topical corticosteroid counseling methods.

Pharmacists reported that verbal counseling (face-to-face) was the most frequently used counseling method (51.2%), followed by verbal and printed information (face-to-face) (39.2%), and the least reported method was a demonstration of the application, which was reported by 1.7% only (Figure 1). The majority of pharmacists believed that the pharmacist’s explanation is the patients’ main source of information about topical corticosteroids (62%), followed by the internet (57.9%) and doctors’ explanations (51.9%), and the least reported was the product information leaflet (28.2%). When asked about the time spent in counseling, pharmacists reported a median of three minutes for both prescription and non-prescription topical corticosteroids, while they reported a median of four minutes to prepare for counseling. Table 3 summarizes the time required for the counseling practices of topical corticosteroids.

**Figure 2:**
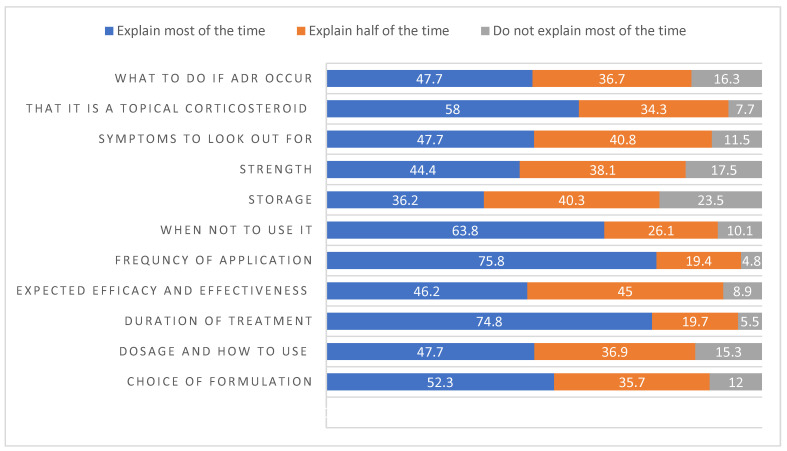
Percentages of counseling practices of topical corticosteroids.

Most of the time, community pharmacists counseled patients on the frequency of application per day and duration of treatment (75.8% and 74.8%, respectively). They also explained that the medication used was a topical corticosteroid (58%), skin conditions where topical corticosteroids should not be used (63.8%), and the choice of formulation for a specific application site (52.3%). Other items that they did not explain most of the time were the expected efficacy and effectiveness, strength, symptoms of an adverse drug (ADR) event, what to do if an ADR occurred, the dose and use methods, and storage instructions (Figure 2). The median counseling practice score was 17, with an IQR of 14–21. The normality assumption was violated with the Shapiro–Wilk test (*p* < 0001) (see Figure S1). Age was associated with a higher counseling practice score, with an adjusted OR (aOR) of 1.09 (95% CI: 1.02 to 1.15); compared to Saudis, non-Saudis had lower practice scores, with an aOR of 0.53 (95% CI: 0.27 to 0.75). The central region had a higher practice score than the eastern region, with an aOR of 1.89 (95% CI: 1.06 to 3.36). Part-time employees had lower practice scores, with an aOR of 0.5 (95% CI: 0.28 to 0.91). Technician degree holders had a lower practice score, with an aOR of 0.2 (0.05 to 0.74), compared to bachelor’s degree holders. Residency holders had higher practice scores, with an aOR of 33 (95% CI: 3.14 to 369.8). See Table S1 for the regression results.

Most community pharmacists reported barriers to counseling (53.5%), with the most common cause being a lack of time (33.7%), followed by patients’ negative perceptions towards topical corticosteroids (30.9%). Regarding patients’ knowledge about topical corticosteroids, most community pharmacists (>50%) did not think that patients had knowledge about their efficacy and effectiveness, strengths, adverse drug effects (ADEs), what to do in case ADEs happened, proper use, and storage.

### 3.3. Adverse Drug Events of Topical Corticosteroids

Most community pharmacists (54%) did not have patients complaining of adverse drug effects from topical corticosteroids. However, most agreed that misuse was the most likely cause of topical corticosteroid adverse drug events (53.7%), followed by medication overuse, such as patient self-treatment (48%). Among the 46% of the community pharmacists who reported patients complaining of adverse drug effects, dry skin, itchiness, and irritation were the most common side effects reported by the community pharmacists (69.4%). Other adverse effects included skin pigmentation and decolorization (47.8%), skin atrophy and stretch marks (40.4%), acne and folliculitis (34%), hot flashes, rosacea, perioral dermatitis, skin infection (32.4%), bruises (24.6%), hirsutism (23.4%), and capillary dilatation (19.9%). Only 11.5% of the community pharmacists reported systemic adverse events, including ocular symptoms.

In the case of adverse events, the majority of community pharmacists reported that they advised patients to discontinue topical corticosteroids and see a doctor (83.5%). Half of the pharmacists reported that they might re-educate patients and recommend a re-trial of the treatment (50%), whereas only 28.5% reported the ADR to the National Pharmaceutical Regulatory Agency (NPRA).

## 4. Discussion

The American Society of Health-System Pharmacists (ASHP) guidelines on pharmacist-conducted patient education and counseling recommend providing the following information during patient counseling: name and description of the medicine, indications, route of administration, dose and dosage form, directions for use, duration of therapy, special directions, precautions, side effects, and contraindications [30]. Our study found that the most common counseling points regarding topical corticosteroids among community pharmacists were the frequency of application per day and the duration of treatment, which were explained most of the time by 75.8% and 74.8% of the participants, respectively. These findings were lower than those reported in a previous study conducted in Malaysia [23]. In that study, the proportion of community pharmacists who reported that they provided information on the frequency of use was 92.1%, and for the duration of use, it was 86.5%. However, the rate of explanation for the duration of therapy in our study was higher than that reported in another study conducted in South Korea [24]. In the study by Kang et al., the duration of therapy was explained most of the time by approximately 53% of the participants, but the frequency of application was still higher than that in our study, with a reported proportion of more than 80%.

The results of this study provide valuable insights into the factors associated with counseling practice scores among healthcare professionals. The finding that older age was associated with higher counseling practice scores suggests that experience and seniority may play a role in developing strong counseling skills. The lower scores observed among non-Saudi and part-time employees point to potential disparities that could be addressed through targeted training and support programs. Interestingly, healthcare workers in the central province exhibited significantly higher practice scores than those in the eastern province, hinting at regional variations that merit further exploration. The lower scores reported by those with only a technician degree, relative to bachelor’s degree holders, underscores the importance of comprehensive education and training in cultivating effective counseling competencies. The substantially higher scores observed among residency holders highlight the value of advanced clinical training in enhancing counseling practices. Overall, these results provide a nuanced understanding of the factors influencing counseling practices, which can inform the development of tailored interventions to improve the quality of patient-centered care.

The most commonly reported barrier to counseling in our study was lack of time, which could partially explain the difference in counseling aspects covered in our study and previous studies. This lack of time could be because of how community pharmacies operate in Saudi Arabia. In addition to dispensing medications, community pharmacies sell non-pharmaceutical products (cosmetics, baby products, etc.) [31]. This might hinder the pharmacist’s ability to spend sufficient time with the patient for counseling, especially during peak hours. Allowing non-pharmacy staff to be responsible for selling non-pharmaceutical products may mitigate this issue.

In our study, the rate of adverse effects reported by the community pharmacists for the patients who used topical corticosteroids differed from those reported in the literature [32]. In a randomized, double-blind, comparative study of the unrestricted, continuous use of 1% pimecrolimus cream versus topical corticosteroids (0.1% triamcinolone acetonide for trunk and limbs and 1% hydrocortisone for face, neck, and intertriginous areas) for one year in 658 adults with moderate to severe atopic eczema, 1% of those applying topical corticosteroids developed striae [32]. The difference in the reported rates of adverse effects when using topical corticosteroids could be due to the fact that these rates were calculated based on patients’ complaints to community pharmacists. This method may be influenced by memory recall and reporting biases. Additionally, the study only considered a percentage of community pharmacists who served hundreds or thousands of patients using topical corticosteroids. As a result, the exact number of ADRs and the total number of patients using topical corticosteroids is unknown, making it difficult to accurately determine the ADR rates.

In our study, the majority of community pharmacists believed that misuse was the primary cause of topical corticosteroid adverse drug events, followed by medication overuse, such as self-treatment by patients. In a previous study, it was found that out of 6723 new patients, 379 (5.63%) were facing misuse and adverse effects of topical corticosteroids, with 78.89% being female. The majority of these patients were between the ages of 10 and 29 years. The main reasons for using topical corticosteroids were to lighten the skin color and to treat melasma and suntan. The most commonly observed adverse effects were acne (37.99%) and telangiectasia (18.99%). It was noted that 88.92% of patients used potent or very potent preparations, including topical combination preparations containing corticosteroids, antibiotics, or antifungal agents [33].

Moreover, pharmacists, paramedical personnel, and patients’ friends and family, as well as general physicians and dermatologists, were found to be responsible for the misuse of topical corticosteroids. This was because they needed to emphasize the adverse effects and proper dosing of these medications to patients. The study suggested a need for more knowledge among medical and paramedical personnel about the appropriate use of topical corticosteroids. It highlighted the need for improvements in continuing medical education programs [33].

Pharmacists have a vital function in preventing the misuse of topical corticosteroids, especially in situations where patients may request inappropriate refills for various reasons. In our study, 62.0% of the community pharmacists believed that patients relied on the pharmacist’s explanation as their primary source of information about topical corticosteroids. Therefore, it is essential to raise awareness about potential issues related to topical corticosteroid misuse by incorporating these topics into the curriculum of pharmacy degree programs. This will help create a community of pharmacists better equipped to promote the responsible dispensing of topical corticosteroids in the future. In addition, 57.9% of the community pharmacists believed that patients used the Internet as their main source of information about topical corticosteroids. Therefore, including topical corticosteroid topics in the school curricula would promote better understanding and awareness, allow pharmacists to contribute to correcting Internet misinformation, and provide patients with accurate and relevant information.

On the other hand, another study suggested that patients may be less satisfied with the counseling provided if they believe the amount of information they receive is excessive. This has been alluded to by health professionals when considering whether to provide patients with lists of side effects in the written medical information [34]. When counseling patients about the adverse effects of topical corticosteroids, pharmacists must distinguish between them and the adverse effects of systemic corticosteroids. In addition, the potency of the topical corticosteroids should be considered. In our study, the potency of the dispensed topical corticosteroids was not reported, preventing the study from finding an association between the adverse effects reported and the potency of topical corticosteroids.

This study had several limitations. First, the survey-based design of the study meant that it was subject to potential biases inherent to self-reported data, such as recall and representational biases. Second, as this was a cross-sectional study, it was not possible to infer causation, and future longitudinal studies are required to confirm these findings. Third, although the study met the needed sample size, its results were only generalizable to practicing pharmacists in Saudi Arabia. Fourth, the study did not determine whether participants searched for information to answer the knowledge section, and the survey completion time needed to be examined.

Future directions in community pharmacy practice regarding topical corticosteroids involve addressing the identified gaps and challenges to improve patient care. One crucial aspect is the need for continuing education and training programs for community pharmacists to enhance their knowledge and counseling skills related to topical corticosteroids. These programs should provide accurate and up-to-date information about the safety, potency, appropriate use, and potential side effects of topical corticosteroids. Additionally, interprofessional collaboration between pharmacists and dermatologists should be promoted to ensure that consistent and evidence-based information is provided to patients. Furthermore, technological advancements can be utilized to develop digital tools and resources that assist pharmacists in counseling patients and providing personalized instructions. By implementing these future directions, community pharmacists can play a crucial role in optimizing the use of topical corticosteroids and improving patient outcomes in managing various skin conditions.

## 5. Conclusions

Pharmacists play a critical role in ensuring that patients correctly apply topical treatment. They are often the final healthcare professionals that patients see, and, as such, they can help patients remember and understand the instructions they have received from doctors and nurses. This can improve treatment outcomes. Community pharmacists, who regularly interact with patients in long-term conditions, are well-positioned to monitor treatment responses and offer advice or reminders about the correct treatment application. Moreover, improving counseling behaviors may involve addressing pharmacists’ attitudes toward providing patient education.

## Figures and Tables

**Table 1:** Baseline characteristics of the surveyed participants (total N = 418).

Variable	Total, N = 418
Age in years, median (IQR)	29 (26–34)
Male, n (%)	139 (33.3%)
Saudi, n (%)	240 (57.4%)
Region of Practice, n (%)	
Eastern Province	108 (25.8%)
Western Province	125 (29.9%)
Central Province	60 (14.4%)
Northern Province	54 (12.9%)
Southern Province	70 (16.7%)
Community Pharmacy type, n (%)	
Chain	353 (84.4%)
Independent pharmacy	65 (15.6%)
Work status, n (%)	
Part-time	37 (8.9%)
Full-time	381 (91.1%)
Level of education, n (%)	
Technician	8 (1.9%)
Bachelor of pharmacy	265 (63.4%)
Doctor of pharmacy	133 (31.8%)
Post-graduate (MS/PhD)	9 (2.2%)
Post-graduate (residency)	3 (0.7%)
Experience as a community pharmacist in years, median (IQR)	3 (2–9)
Monthly dispensed prescriptions of topical steroids, median (IQR)	30 (10–50)
Monthly dispensed non-prescriptions of topical steroids, median (IQR)	25 (10–50)

**Table 2:** Frequency of dispensed topical steroids based on the type in the pharmacy (total N = 418).

Frequency	High Frequency, n (%)	Moderate Frequency, n (%)	Low Frequency, n (%)	Minimal Frequency, n (%)
Steroid-only agents (e.g., DermAid 1% hydrocortisone cream, Elomet cream 0.1%)	177 (42.3)	147 (35.2)	56 (13.4%)	38 (9.1%)
Steroid–antibiotics or steroid–antifungals combination agent (e.g., Fucicort cream, Daktacort cream)	141 (33.7%)	163 (39%)	66 (15.8%)	48 (11.5%)
Steroid–keratolytic agent (e.g., Beprosalic ointment)	60 (14.4%)	121 (28.9%)	128 (30.6%)	109 (26.1%)
Steroid–other ingredients combination agents (e.g., Daivobet ointment)	76 (18.2%)	106 (25.4%)	131 (31.3%)	105 (25.1%)

**Table 3:** Time required for counseling practices of topical corticosteroids.

Task	Median Time (IQR)
Counseling Preparation time *	4 (2–5)
Time spent in face-to-face (verbal) counseling	
Minutes per patient on **prescription** topical corticosteroids	3 (2–5)
Minutes per patient on **non-prescription** topical corticosteroids	3 (2–5)

* “Counseling preparation time” refers to the time invested in searching, reviewing, evaluating, and organizing information.

## Data Availability

All the data of this research have been presented in this paper; however, the raw data are available upon request from the corresponding author (Kurdi SM).

## Supplementary Materials

The following supporting information can be downloaded at: https://www.mdpi.com/article/10.3390/healthcare12141425/s1, Figure S1: Median of counseling practice score was 17 with IQR of 14–21; Data was far from normal with Shapiro wilk test (*p* < 0001); Table S1: Model CoefficientsPractice_score_ordinal.
