# Supplementary material for: Insight into Current Practices of Community Pharmacists in Topical Corticosteroid Prescribing and Counseling: Cross-Sectional Survey Study from Saudi Arabia
Source: Healthcare (Basel). 2024 Jul 17;12(14):1425. doi: 10.3390/healthcare12141425 (PMC11276632; doi:10.3390/healthcare12141425)
Supplement: Supplementary file 1 [file healthcare-12-01425-s001.zip › healthcare-3042641-supplementary.pdf]

Supplementary Metarials

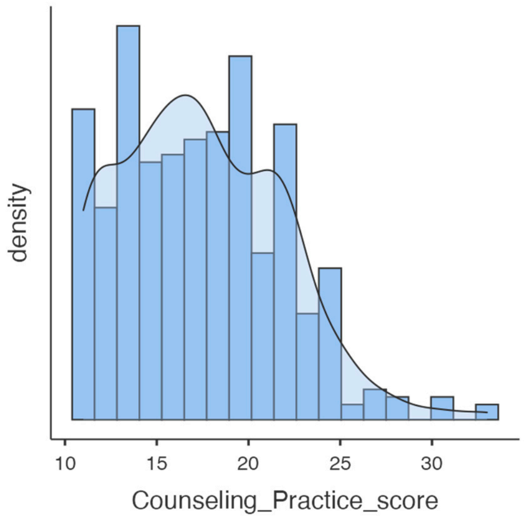

Figure S1: Median of counseling practice score was 17 with IQR of 14 – 21. Data was far from normal with Shapiro wilk test (P<0001).

Table S1: Model Coefficients - Practice_score_ordinal

						95% Confidence Interval	
**Predictor**	**Estimate**	**SE**	**Z**	**p**	**Odds ratio**	**Lower**	**Upper**
age	0.0813	0.0296	2.751	0.006	1.085	1.0244	1.151
sex:							
Female – Male	0.2298	0.2121	1.084	0.279	1.258	0.8303	1.908
nationality:							
Non-Saudi – Saudi	-0.7912	0.2587	-3.059	0.002	0.453	0.2727	0.753
region:							
Western – Eastern	0.4290	0.2396	1.791	0.073	1.536	0.9606	2.458
Central – Eastern	0.6348	0.2926	2.170	0.030	1.887	1.0642	3.355
Northern – Eastern	0.1075	0.3079	0.349	0.727	1.114	0.6096	2.041
Southern – Eastern	-0.3807	0.2751	-1.384	0.166	0.683	0.3979	1.171
pharmacy_type:							
Independant – Chain	-0.2812	0.2553	-1.102	0.271	0.755	0.4576	1.246
work_status:							
Full-time – Part-time	-0.6786	0.3013	-2.253	0.024	0.507	0.2800	0.914
Experience_years	-0.0562	0.0332	-1.690	0.091	0.945	0.8849	1.008

Table S1: Model Coefficients - Practice_score_ordinal

						95% Confidence Interval	
**Predictor**	**Estimate**	**SE**	**Z**	**p**	**Odds ratio**	**Lower**	**Upper**
education_level:							
Technicians – BPharm	-1.6156	0.6824	-2.367	0.018	0.199	0.0492	0.741
PharmD – BPharm	-0.2889	0.2172	-1.330	0.184	0.749	0.4890	1.146
MS/PhD – BPharm	-0.3612	0.5785	-0.624	0.532	0.697	0.2219	2.202
Residency – BPharm	3.4829	1.1779	2.957	0.003	32.554	3.1455	369.812

The survey

Community pharmacists knowledge practices

	Item	Information		Code/ Score
**A. Demographic characteristics, dispensing and sales of topical corticosteroids**				
**1**	Age (years)			
**2**	Gender	Female		1
		Male		2
**3**	Nationality	Saudi		1
		Non-Saudi		2
**4**	Current workplace	Community Pharmacy		1
		Hospital pharmacy		2
		Other:		2
**4a**	Type of community pharmacy	Chain pharmacy		1
		Independent pharmacy		2
**4b**	Type of Hospital	Private hospital		1
		Governmental hospital		2
**4c**	Work status	Part-time		1
		Full-time		2
**5**	Experience as a community pharmacist or outpatient pharmacist (years)	(years)		
	Level of education	Bachelor of pharmacy		1
		Doctor of pharmacy		2
		Post-graduate (MS/PhD)		3
		Post-graduate (Residency)		4
**7**	Have you ever been trained in reporting adverse drug events?	Yes		1
		No		2
**8**	What is the % of total sales of non-prescription TCS at your pharmacy on average per month?	Non-prescription (%)	X	
		Prescription (%)	100 − X	
		**Total**	**100%**	
**9**	What is the % of sales by recommendation of non-prescription TCS at your pharmacy on average per month?	Patient self-selection (%)	Y	
		Pharmacist recommendation (%)	100 − Y	
		**Total**	**100%**	
**10**	Please rate the following non-prescription over-the-counter topical	Steroids only agents (e.g. DermAid 1% Hydrocortisone cream, Elomet Cream 0.1%)		

	corticosteroids in order of sales frequency<br><br>**(Note give 1 to the most frequently sold agents and 4 to the least frequently sold)**	Steroid-antibiotics or steroid antifungals combination agent (e.g. Fucicort cream, Daktacort cream)		
		Steroid-keratolytic agent (e.g. Beprosalic ointment)		
		Steroid-other ingredients combination agents (e.g. Daivobet ointment)		

**B. Patient Counselling on the Use of Topical Corticosteroids**				
**11**	Please answer the following questions about your patient counselling practices on the use of topical corticosteroids			
**11a**	The most frequently used counselling method	Printed Information		
		Face-to-face (verbal)		
		Face-to-face (verbal and printed)		
		Demonstration of Application		
**11b**	Counselling Preparation time<br><br>"Counselling preparation time" refers to the time invested in searching, reviewing, evaluating, and organizing information, including verifying prescription and checking patients past medical history before counselling a patient.	On average minutes per patient		
**11c**	Time spent in face-to-face (verbal) counselling	On average minutes per patient on **prescription** topical corticosteroids		
		On average minutes per patient on **non-prescription** topical corticosteroids		

**12**	Please mark on the items below to indicate to what extent you counsel patients using topical corticosteroids			
		**Explain most of the time (2)**	**Explain half the time (1)**	**Do not explain most of the time (0)**
**12a**	That it is a topical corticosteroid			
**12b**	Expected efficacy and effectiveness			
**12c**	Skin conditions and diseases where topical corticosteroids should not be used			

	E.g.: do not use on burns			
**12d**	Strength (potency)			
**12e**	Adverse drug events			
**12f**	Symptoms to look out for in an adverse drug event			
**12g**	How to use- dosage<br>E.g.: **fingertip unit**: amount of ointment or cream squeezed out of a tube, from the tip of an adult index finger to the first crease in the finger, which may be sufficient for a diseased site of two adult-hand size			
**12h**	How to use- **frequency** of application in a day			
**12i**	How to use- **duration** of treatment			
**12j**	How to use- **choice of formulation** (ointment, cream, lotion, etc.) for specific application site			
**12k**	Precautions for storage and application of leftover topical corticosteroids after treatment completion<br>E.g.: do not remove medication from its original packaging and transfer to a separate container, do not use remaining medication in other conditions at patients' own discretion			
**Questions 13-15 are regarding your counselling practices to assist patient self-treatment with non-prescription over-the-counter topical corticosteroids**				
**13**	In which of the following cases would you recommend a patient seeking non-prescription over-the-counter topical corticosteroids to see a doctor? (Multiple selection allowed)	Skin lesions with effusion		1
		Skin conditions of moderate or greater severity		1
		Signs of skin infection		1

		Neonates and infants (less than 24 months old) (excluding nappy rash)		1
		Applying topical corticosteroids on a large area of body surface		1
**14**	For how many **days** do you tell patients on non-prescription over-the-counter topical corticosteroids that it is okay to use continuously?	Maximum days		
**15**	Do you believe that there are **barriers** to counselling patients on topical corticosteroids?<br><br>(“No”, to move on to Question 17)	Yes		1
		No		0
**15a**	Please select the barriers to counseling (multiple selection allowed)	Lack of counselling material		
		Lack of time for counselling		
		Doctor’s negative perception towards pharmacists’ counselling		
		Patients’ negative perception towards topical corticosteroids		
		Presume patients already know well about topical corticosteroids		

**C. Pharmacists’ perception on patients’ knowledge on topical corticosteroids prior to counselling**				
**16**	Do you think patients have adequate knowledge on the following aspects of topical corticosteroids?			
		Yes	No	
**16a**	That it is a topical corticosteroid			

**16b**	Efficacy and effectiveness			
**16c**	Strength (potency)			
**16d**	(Patients on **non-prescription** topical corticosteroids) Adverse drug events			
**16e**	(Patients on **prescription** topical corticosteroids) Adverse drug events			
**16f**	What to do when an adverse drug event occurs			
**16g**	Know how to use- **(dosage, treatment duration, frequency of application, choice of formulation** for specific application site) and to comply with it			
**16h**	Know how to store and apply leftover topical corticosteroids after treatment completion and comply with it<br><br>E.g. do not use remaining medication on other conditions at patients' own discretion			
**17**	Please select **three** options from the given choices that you believe are patients' main source of information about topical corticosteroids	Pharmacist's explanation		1
		Doctor's explanation		2
		Product information leaflet (PIL)		3
		Internet		4

**D. Adverse drug events of topical corticosteroids**			
**18**	Have you had patients complaining (including visits and phone calls) of adverse drug events after using TCS at your pharmacy	Yes	1
		No	0
**19**	Please rank the factors you think are the causes of adverse drug events of TCS	Medication misuse (e.g. Use on not applicable conditions)	
		Medication characteristics (e.g. Strength/ potency)	

	Please rank the factors from 1 to 4, give “1” to the most likely factor	Patient characteristics (e.g. Elderly, with chronic diseases, neonates and infants)	
		Medication overuse (e.g. Patient self-treatment, not complying with treatment instructions)	
**20**	Please select three (3) adverse drug events of TCS that patients frequently complain	Dry skin, itchiness, irritation	
		Bruises	
		Acne, folliculitis	
		Hirsutism (excessive hair growth)	
		Systemic adverse events (including ocular symptoms)	
		Capillary dilatation	
		Skin atrophy, stretch marks	
		Chang in skin colour (decolourisation, pigmentation)	
		Hot flashes, rosacea, perioral dermatitis	
		Skin infection (bacterial, fungal, viral)	
**21**	What do you do when patients on topical corticosteroid complain of adverse drug events?<br><br>Multiple selection allowed	Discontinue and recommend seeing a doctor	
		Report to National Pharmaceutical Regulatory Agency (NPRA)	
		Check if the patient has been using topical corticosteroids as directed. Re-educate patient and recommend re-trial of the treatment	
**22**	Please rank the below three topical corticosteroids according to their strength (potency)<br><br>Rank TCS strength (potency) from 1-3, give "1" to the highest strength (potency) agent, and give "3" to the lowest strength (potency)	Derm-Aid 1% (Hydrocortisone) ointment	
		Elomet (Mometasone furoate) 0.1% cream	
		Dermovate (clobetasol propionate) 0.05% ointment	
